# A randomized wait-list control trial to evaluate the impact of a mobile application to improve self-management of individuals with type 2 diabetes: a study protocol

**DOI:** 10.1186/s12911-016-0381-5

**Published:** 2016-11-15

**Authors:** Laura Desveaux, Payal Agarwal, Jay Shaw, Jennifer M. Hensel, Geetha Mukerji, Nike Onabajo, Husayn Marani, Trevor Jamieson, Onil Bhattacharyya, Danielle Martin, Muhammad Mamdani, Lianne Jeffs, Walter P. Wodchis, Noah M. Ivers, R. Sacha Bhatia

**Affiliations:** 1 Women’s College Hospital Institute for Health Systems Solutions and Virtual Care, Women’s College Hospital, 76 Grenville Ave, Toronto, ON M5S 1B2 Canada; 2 Women’s College Research Institute, Women’s College Hospital, 76 Grenville Ave, Toronto, ON Canada; 3 Department of Psychiatry, Women’s College Hospital and University of Toronto, Toronto, ON Canada; 4 Division of General Internal Medicine, St. Michael’s Hospital, 209 Victoria St, Toronto, ON Canada; 5 Li Ka Shing Centre for Healthcare Analytics Research and Training, St. Michael’s Hospital, 209 Victoria St, Toronto, ON Canada; 6 Li Ka Shing Knowledge Institute, St. Michael’s Hospital, 30 Bond St, Toronto, ON Canada; 7 Institute for Clinical Evaluative Sciences, 2075 Bayview Ave, Toronto, ON Canada; 8 Insititue for Health Policy, Management, and Evaluation, University of Toronto, 155 College St, Toronto, ON Canada; 9 Department of Family and Community Medicine, Women’s College Hospital and University of Toronto, 76 Grenville Ave, Toronto, ON Canada; 10 Department of Medicine, University of Toronto, Toronto, ON Canada; 11 Toronto Rehabilitation Institute, 550 University Avenue, Toronto, ON Canada

**Keywords:** E-Health, Randomized controlled trial, Diabetes, Self-management, Implementation science, Mobile health

## Abstract

**Background:**

Management of diabetes through improved glycemic control and risk factor modification can help prevent long-term complications. Much diabetes management is self-management, in which healthcare providers play a supporting role. Well-designed e-Health solutions targeting behavior change can improve a range of measures, including glycemic control, perceived health, and a reduction in hospitalizations.

**Methods:**

The primary objective of this study is to evaluate if a mobile application designed to improve self-management among patients with type 2 diabetes (T2DM) improves glycemic control compared to usual care. The secondary objectives are to determine the effects on patient experience and health system costs; evaluate how and why the intervention worked as observed; and gain insight into considerations for system-wide scale-up. This pragmatic, randomized, wait-list-control trial will recruit adult participants from three Diabetes Education Programs in Ontario, Canada. The primary outcome is glycemic control (measured by HbA1c). Secondary outcomes include patient-reported outcomes and patient-reported experience measures, health system utilization, and intervention usability. The primary outcome will be analyzed using an ANCOVA, with continuous secondary outcomes analyzed using Poisson regression. Direct observations will be conducted of the implementation and application-specific training sessions provided to each site. Semi-structured interviews will be conducted with participants, healthcare providers, organizational leaders, and system stakeholders as part of the embedded process evaluation. Thematic analysis will be applied to the qualitative data in order to describe the relationships between (a) key contextual factors, (b) the mechanisms by which they effect the implementation of the intervention, and (c) the impact on the outcomes of the intervention, according to the principles of Realist Evaluation.

**Discussion:**

The use of mobile health and virtual tools is on the rise in health care, but the evidence of their effectiveness is mixed and their evaluation is often lacking key contextual data. Results from this study will provide much needed information about the clinical and cost-effectiveness of a mobile application to improve diabetes self-management. The process evaluation will provide valuable insight into the contextual factors that influence the application effectiveness, which will inform the potential for adoption and scale.

**Trial registration:**

Clinicaltrials.gov NCT02813343. Registered on 24 June 2016 (retrospectively registered).

Trial Sponsor: Ontario Telemedicine Network

## Background

E-Health solutions are increasingly common in healthcare, and involve the use of an electronic device or monitoring system by individuals or their healthcare providers to monitor or improve their health status [1]. Well-designed e-Health solutions for diabetes management targeting health behaviour change can improve a range of measures, including improvements in glycemic control, perceived health, and a reduction in hospitalizations [2, 3]. Furthermore, e-Health has been shown to streamline communication among providers and enable the provision of care by providing providers with improved knowledge, skills, and confidence [4]. Most notably, these solutions may provide an opportunity to improve access to enhanced care for marginalized populations and may be cost-effective to implement at scale.

An obvious area in which e-Health holds potential is the management of Type 2 Diabetes (T2DM). The global prevalence of diabetes continues to rise, with projections estimating 9 % of the population will be impacted by 2035 [5]. In Canada alone, over 2.4 million individuals are living with diabetes, with anticipated increase in prevalence to 3.7 million Canadians by 2018 [6]. Diabetes is the leading cause of adult blindness [7] and end stage renal disease among Canadians [8], with the cost of care for individuals with diabetes currently reaching 3–4 times that of those without the disease [6].

Management of diabetes through improvements in glycemic control [9] and risk factor modification can help prevent long-term complications [10]. Unfortunately, evidence-to-practice gaps are well documented in diabetes care [11]. In addition, much of the management of diabetes lies not in the hands of health care providers, but in the day-to-day self-management of the millions of people living with this chronic condition whose lives are complex and not solely defined by the disease. This reality drives the increasing interest in innovative strategies to improve self-management that emphasizes convenience and recognizes the realities of people’s busy lives. Although the use of mobile interventions to improve diabetes self-management significantly improved glycaemic control [12], substantially heterogeneity in intervention design and implementation continues to be a reality [3]. Among successful interventions, the most common behaviour change techniques include prompt self-monitoring and feedback on performance [3]- features that are easily incorporated as part of mobile technology.

In light of the evidence, health system decision makers in Ontario, Canada are interested in evaluating the effectiveness, implementation, and impact of mobile-based self-management tools for T2DM among diverse patient populations throughout the province. Lessons learned are likely to be useful across a wide variety of chronic conditions and therefore have significant potential to address the triple aim of improved population health outcomes, enhanced patient experience and reduced health care costs per capita. This pragmatic evaluation will explore not only whether the intervention produces an effect, but how and why this effect (or lack thereof) is produced.

The primary objective of this study is to evaluate whether a mobile application designed to improve self-management and experience of care can improve clinical outcomes compared to usual care for people with T2DM. The secondary objectives are to determine the effects on patient experience and health system costs; to evaluate how and why the intervention worked as observed; and to gain insight into considerations for system-wide scale-up. The inclusion of the embedded process evaluation within the overall study design presents a novel contribution in the e-Health literature by including a methodological approach to provide insight into how e-Health tools function more generally, beyond their application to diabetes care. The project evaluation is based on a model of integrated knowledge translation [13], whereby the health system decision makers, implementation leads, and scientific evaluators have collaborated to inform the development of the research project from the outset.

## Methods

We designed a pragmatic, randomized, wait-list-control trial with an embedded qualitative process evaluation to evaluate whether and how a mobile application designed to improve self-management and experience of care among patients with T2DM has an effect on clinical outcomes and healthcare utilization. The protocol received ethics approval from Research Ethics Boards at participating institutions, including Women’s College Hospital, St. Joseph’s Care Group, North York General Hospital, and William Osler Health System. The trial is registered on ClinicalTrials.gov (NLM Identifier: NCT02813343).

### Setting

Most health services in Ontario, Canada are financed through the publicly-funded Ontario Health Insurance Program (OHIP). OHIP covers medically necessary services provided by physicians, including primary care, emergency services, inpatient medical services, and specialist visits. In addition to OHIP, the Ministry of Health & Long-Term Care funds Diabetes Education Programs (DEPs), which are community-based, multi-disciplinary programs accessible through both physician and self-referral. DEPs provide diabetes education services for clients who are aged 18 or over. Their primary focus is the provision of diabetes education and management services for individuals diagnosed with diabetes or pre-diabetes according to the Canadian Diabetes Association Guidelines [14]. This includes education around self-monitoring blood glucose levels, screening for complications, nutrition, physical activity, and appropriate management strategies including vascular protection, and optimizing glycemic control.

Participating DEPs were selected by the Ontario Telemedicine Network (OTN) and include the Diabetes Health Centre in Thunder Bay, the Diabetes Education Center at North York General Hospital, and two Diabetes Education Centers belonging to the William Osler Health System. OTN reached out to the Provincial Chronic Disease Management-Local Health Integration Leads Council for help in identifying organizations who would be good partners in this initiative. These sites serve a socially and ethnically diverse range of patients with diabetes, representing more than 4500 Ontarians annually. Each site serves distinct populations, including a large aboriginal population in Thunder Bay and visible minorities and new immigrants in the William Osler Health System.

### Stakeholders

The intervention will be implemented by the OTN, a non-profit, government funded organization and the largest provider of telemedicine services in the province of Ontario [15]. OTN’s involvement in an advisory capacity increases the likelihood that the study results will have a future impact on health system policy. This engages the principles of integrated knowledge translation to collaboratively produce knowledge [16], with the aim of optimizing health care delivery and associated clinical outcomes. The study is funded by Canada Health Infoway.

### Trial design

**Fig. 1:**
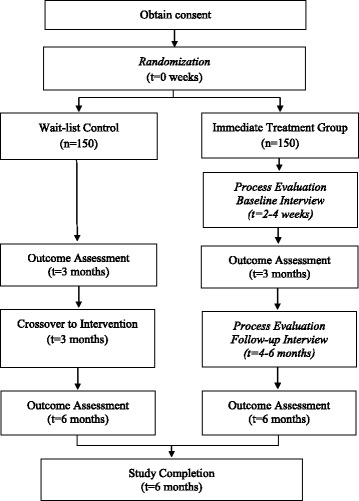
Flow of Participants

The study is a pragmatic, randomized, wait-list-control trial with blinded outcome assessment. It is important to note that the study design reflects an external, third-party evaluation. The evaluation team is independent from both the application vendor and the implementation team, which is coordinated and controlled by OTN. A pragmatic randomized trial design was chosen because it is a methodology that guides the assessment of whether an intervention works when introduced into a public health or clinical setting (i.e., in real-life conditions). Participants are randomized into one of two groups: an immediate treatment group (ITG) and a wait-list-control (WLC) group. The ITG group immediately begins using the application (e-Health intervention), for a total duration of six months (refer to Fig. 1). The WLC group receives usual care for the first three months, at which point they will cross over to the intervention arm and use the application for a total of 3 months.

#### Eligibility

Patients are eligible for inclusion into the study if they meet the following criteria: 1) adults over the age of 18; 2) obtaining care for T2DM at a participating DEP; 3) HbA1c ≥ 8.0 % (and at least 1 % above the participant’s target level) on most recent laboratory report within the last 3 months 4) currently using an active email address or able and willing to obtain one; and 5) able to read the English language (self-reported). Patients are excluded if they have Type 1 diabetes; are on continuous glucose monitoring; have an insulin pump; are on dialysis; are pregnant; or are unable to use a computer or mobile phone due to severe mental or physical impairment. The inclusion criteria are intentionally broad to align with the objectives of pragmatic trial design to determine whether the application works under usual conditions to promote generalizability [17]. The diagnostic exclusion criteria reflect a set of conditions in which there are distinct and unique self-management needs, which the application is not currently designed to address.

#### Recruitment

**Fig. 2:**
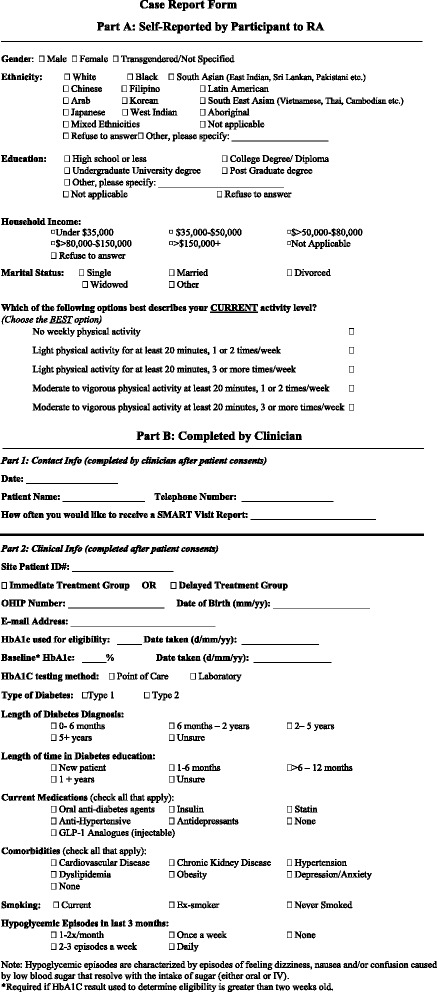
Case Report Form

Participants are recruited from participating DEPs associated with three institutions across Ontario. A clinician at each site identifies potential eligible participants during their regularly scheduled clinical appointment. Interested individuals meet with a site coordinator who provides them with an information brochure and a consent form to review. Interested participants contact the research assistant using the dedicated study phone line. The research assistant obtains and documents verbal informed consent and collects baseline outcome measures (refer to Fig. 2, Part A). Participants are then randomized to one of the study arms. Following randomization, the site coordinator collects clinical information (refer to Fig. 2, Part B) and provides participants in the ITG with a study-specific mobile phone. Participants enter initial clinical information into the application (medications, etc.) while in clinic and the data is reviewed for accuracy by a clinician (nurse, nurse practitioner, or certified diabetes educator). Participants in the WLC group receive their application-enabled phone in person following completion of the control phase (three months after enrollment).

OTN has partnered with Samsung to provide a total of 300 smartphones with the mobile health application pre-loaded. Each device will be connected to the Telus mobile network in addition to having the ability to connect to wireless networks. As all phones need to be distributed as part of the partnership, a targeted sample of 300 patients will be recruited.

#### Allocation

Randomization is centralized, web-based and generated by the Applied Health Research Centre (AHRC) at the Li Ka Shing Knowledge Institute of St. Michael’s Hospital. Randomization is stratified by site using randomly permuted blocks of varying sizes, which are not known by the study team.

Once the participant has been enrolled and the baseline assessments are completed, randomization occurs in REDCap™, a web-based electronic data entry system, where the participant is automatically assigned a group allocation (ITG or WLC).

#### Interventions

##### Mobile Application (E-Health Intervention)

The intervention is a commercially available mobile application that is designed to serve as a virtual coach for patients with T2DM. The application allows participants to enter a range of baseline clinical information, in addition to ongoing data related to diabetes management, including blood glucose values, daily medications, food intake, and activity levels (https://www.bluestardiabetes.com/). The application analyzes inputted data to provide tailored messaging to ‘coach’ participants with respect to their diabetes management. Coaching is a process of continuous improvement involving education regarding targets, negotiating a plan of action to achieve the target, and subsequent monitoring of the patient’s progress toward achievement of the target [18, 19]. Lifestyle coaching empowers participants by giving them the knowledge and skills to work in partnership with providers to achieve health goals [18, 19]. The mobile application has been shown in other contexts and settings to be effective at teaching patients with T2DM about dietary impacts on their blood glucose levels, encouraging patients to generate higher-quality blood glucose data for enhanced self-monitoring, and to improve glycemic control as represented by reduction in levels of HbA1c [20, 21].

The intervention is believed to improve disease control through two patient-mediated processes: tailored messaging and education and facilitated relay of information from the patient to their healthcare provider [22]. The application provides customized, real-time, evidence-based messages based on the Trans-Theoretical Model of Behaviour Change [23] that impact patient motivation, behaviour and education. Educational messages are organized along the American Association of Diabetes Educators 7 Standards of Care [24] and are supported by a library of educational videos that patient can access at any time. The application also facilitates the transfer of disease monitoring data to their primary care provider via a Smart Visit report. The application reminds participants to print this document and bring it to clinical appointments, although the option is available to print the report on demand.

##### Usual Care

Usual care at DEPs are intended to provide multi-disciplinary self-management support to patients with diabetes and may include interactions with a diabetes educator (nurse, nurse practitioner, dietician, pharmacist) or physician specializing in diabetes care. These clinics are designed to support the efforts of the primary care provider, and are not intended to be a substitution for this ongoing relationship. The duration of a patient’s affiliation with a DEP can be highly variable based on clinic, patient and disease factors.

#### Data collection

Baseline information including demographics and health status will be collected using an electronic case report form (CRF) (refer to Fig. 2). HbA1c values will be recorded at baseline, 3 months, and 6 months. Participants who do not have a recent value within two weeks of the study assessment will be given a requisition to perform blood work during the first study visit. Baseline information will be collected by a research assistant via a telephone call, after which the participant will be formally enrolled in the study. Participants who fail to complete the baseline assessment will not be enrolled. Follow up data will be collected using a secure online questionnaire administered via REDCap™ that can be completed by participants from any device with an internet browser and connection (computer, tablet etc.). Weekly reminders will be sent to participants who fail to complete the questionnaire, with a maximum of three reminders. Participants who express a preference for phone communication will be contacted by the research assistant, following the same reminder protocol. Application utilization data is tracked directly through the application and will be extracted at the end of the study.

Healthcare utilization and cost outcome measures will be obtained from health administrative data available through the Institute for Clinical Evaluative Sciences (ICES). At the time of study enrolment, patients will be asked to provide consent to link their OHIP number to health administrative data. ICES is a non-profit research institute with a secure and expansive repository of Ontario’s health-related administrative data from hospital, physician, long term care and other related health services paid for by the provincial health insurance plan. These data are linked using encrypted identifiers. Data from this study will be linked to ICES data using encrypted health card identifiers created by ICES authorized personnel. ICES is a prescribed entity and data custodian under the Personal Health Information Protection Act (PHIPPA).

#### Outcomes

**Fig. 3:**
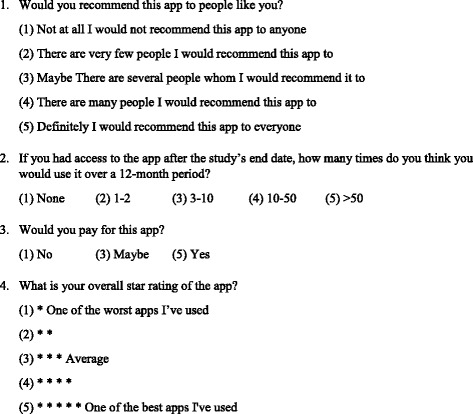
Modified Mobile Application Rating Scale

The primary outcome measurement is glucose control, measured by HbA1c at 3 months. Secondary outcomes which will be evaluated at 3 months are patient-reported outcomes measures (PROMs) and patient-reported experience measures (PREMs), which include data on health system utilization using composites of items from: the Commonwealth Fund Survey for Patients with Complex Needs; the Canadian Institutes of Health Research Community Based Primary Health Care Survey; and the EQ5D [25]. Self-efficacy will be measured using two validated scales: the Problem Areas in Diabetes 5 (PAID 5) [26] and the Summary of Diabetes Self-care Activities (SDSCA-6) [27]. All outcomes will be assessed again at 6 months. Intervention usability is an additional secondary outcome which will be evaluated at the end of study as measured by an adapted version of the Mobile App Rating Scale [28] (refer to Fig. 3 for adapted version) and application utilization data collected routinely collected through the application. Utilization measures include the mean number of engagements per week; mean length of time engaging with the application per week; and the frequency of use of each feature per week (e.g., educational tools, Smart Visit Report, blood sugar monitoring).

In this study, costs of healthcare utilization are used to measure resource intensity. Table 1 outlines the databases used to assess these costs, which will be used to determine frequency and intensity of healthcare utilization and overall individual costs using methods developed for use with Ontario data [29]. Costs of health care resource use will be quantified for patients with algorithms developed for patient-level costing using health administrative data and implemented at ICES [29]. Costs for each encounter that generated an encounter-specific payment (e.g., prescriptions, fee-for-service physician visits) are measured as the fee paid for the service. Costs for hospital encounters are determined using the appropriate resource intensity weight for that particular care setting and the weighted cost derived based on Ontario spending. Costs for long-term care are measured as a fixed per diem based on prevailing government payment rates. Emergency department physicians receive substantial alternative payments that are not visit-related, and the algorithms also ascribe those payments, generally on an average per-patient approach. Capitation payments are calculated based on the payment rate and the particular model of primary care for each patient’s physician in each month of the study period. Team-based payments for family health teams and physician bonus payments for pay-for-performance are not ascribed to individual patients and thus are not included in the analysis.

**Table 1:** Databases and cost components used in the calculation of health system costs before, during, and after cancer treatment

Database	Description	Cost component
Ontario Health Insurance Plan and (OHIP)	Contains claims paid by OHIP for services provided by all eligible health care providers, including physicians, groups, and laboratories.	• Outpatient physician visits • Laboratory services • Non-physician services
National Ambulatory Care and Reporting System	Contains data from hospital- and community-based ambulatory care services, including day surgery, outpatient clinics, and emergency departments.	• Emergency department visits • Dialysis visits • Oncology clinic visits
Discharge Abstract Database	Contains information on patient separations, notably: • clinical data (diagnoses, procedures) • administrative data (institution information, admission type, length of stay, disposition) • resource consumption, defined using case-mix groups and resource intensity weight	• Inpatient hospitalizations
Client Agency Program Enrolment	Registry of patients enrolled in a primary care model. Data elements include program type (family health team, family health organization, family health network, etc.) and patient enrolment status.	• Capitation costs
Ontario Drug Benefit and (ODB)	Contains claims for prescription drugs covered under the ODB program. Primarily includes drug claims for individuals 65 years of age and older, but also coverage under special ODB programs.	• Medication use
National Rehabilitation and Reporting System	Contains client data from adult inpatient rehabilitation facilities, such as • administrative data (referral, admission, and discharge); • health and functional characteristics; • activities and participation (activities of daily living, communication level, social interaction); and • intervention information.	• Rehabilitation admissions
Continuing Care and Reporting System	Contains information about residents receiving facility-based continuing care services. Range of services includes complex continuing care, extended or chronic care, and residential care providing nursing services (that is, long-term care).	• Complex continuing care admissions and long-term care
Home Care Database	Captures information on all services provided or coordinated by Ontario Community Care Access Centres, including client data, intake and assessment information, admission and discharge, diagnosis and procedures, and care delivery.	• Home care services
Ontario Mental Health and Reporting System	Contains data on patients in adult designated inpatient mental health beds in acute and psychiatric facilities. Data elements include admission and discharge information, diagnoses, service utilization or intervention and procedures.	• Mental health admissions
Assistive Devices Program	Contains data on Ontario residents with long-term disability receiving personalized assisted devices to support basic needs, such as insulin pumps and supplies, home oxygen, and respiratory and ventilator equipment.	• Assistive devices

#### Data analysis

All data will be analyzed according to an intention to treat principle. Patient characteristics and baseline HbA1c levels will be summarized using descriptive statistics, including means, quartiles and standard deviations for continuous variables and proportions for categorical variables. The primary outcome, HbA1c levels between treatment groups, will be analyzed using an analysis of covariance (ANCOVA) with follow-up at three months. PROMs and PREMs will be analyzed using an ANCOVA evaluated at 3 months as well. Continuous secondary outcomes including emergency department visits, physician visits, and hypoglycemic episodes will be analyzed using Poisson regression predicted with the two treatment groups. Ordinal categorical variables including medication adherence and frequency of glucose monitoring will be analyzed using an ordinal regression model for the two treatment groups. We will use regression analyses to separately estimate the difference in healthcare costs between the intervention and control groups and include any covariates that are observed to be different between groups in baseline comparisons. To identify the regression model that fits best with the cost data and potential skew, we will follow the steps suggested by Manning and Mullahy [30] to decide between regression on transformed cost data or generalized linear models.

Six-month follow-up data for the immediate treatment group will be analyzed descriptively. In addition, HbA1c levels will be compared at the 6-month point between the two treatment groups using an ANCOVA which will control for baseline HbA1c levels. Application utilization data will be analyzed descriptively, including time (mean duration of use), general frequency of use (mean uses per week), and frequency of use for each application feature. Subgroup analyses based on site and patient demographics, including age, sex, ethnicity, education, and income [31], will also be conducted. All statistical analyses will be performed using R version 3.0.3 (The R Foundation for Statistical Computing; Vienna, Austria).

#### Power calculation

Power was determined assuming an ANCOVA analysis with an estimate correlation between baseline and follow-up HbA1c measurements of 0.80. The power to detect a difference of 0.7 % [32] in HbA1c levels using a standard deviation of 2 % [32] between treatment groups at 3 months is 0.997 with an alpha of 0.05 based on a sample size of 255 (which assumes a dropout rate of 15 % from the target sample size of 300 participants).

### Embedded process evaluation

Many effective health services interventions fail to be widely adopted or translated into real-world practice [33, 34]. Given that innovations in health services are inevitably complex interventions, “an understanding of the causal assumptions underpinning the intervention and use of evaluation to understand how interventions work in practice are vital in building an evidence base that informs policy and practice” ^35 (p.1)^. The UK Medical Research Council’s Guidelines for Process Evaluation of Complex Interventions [35] provides a framework for such evaluations and has directly informed the current evaluation.

The embedded process evaluation involves a qualitative approach using observation and individual semi-structured interviews to evaluate how and why the intervention achieved the effects observed, if any. Using an embedded single case design with cross case synthesis, the objective of the process evaluation is to gain an in-depth understanding of intervention fidelity, the mechanisms of action, and the conditions and factors associated with implementation of the intervention. We will conduct observations of the initial education and training provided to healthcare providers around the purpose of the application and how to interact with it. Qualitative observations will involve a researcher sitting in on the training and taking detailed notes regarding when and how the application is introduced, questions asked, and how participants interact with the technology during the training session [36]. Two observations of 1–2 h in length will take place at each health care site with the aim of capturing the processes and strategies used to introduce the application to patients. Notes will record details around how the application is described to patients/caregivers, any questions asked by patients/caregivers, and the actions taken to explain the use of the application or to practice using the technology itself (where feasible).

To evaluate intervention fidelity and understand the mechanisms of action, we will interview individuals from each implementation site, guided by the principles of Realist Evaluation. Realist Evaluation seeks to understand *what works, for whom, under what circumstances* [37], and will provide crucial insight into the contextual conditions in each clinical and regional setting that support or hinder the success of the intervention in each specific patient group. In order to capture a range of perspectives impacting the implementation of the intervention, we will seek to interview participants and their caregivers, as appropriate; healthcare providers who interact with study participants and the application; organizational administrators who oversee the implementation process at each site; and health system decision makers involved in the implementation of e-Health initiatives in Ontario.

#### Recruitment

##### Participants and caregivers

Participants will be informed of the qualitative process evaluation at the time of enrollment. A sub-sample of participants who indicate interest will be invited to participate in the semi-structured interviews. Participants will be selected to maximize sampling variation, including baseline HbA1c, socioeconomic status, ethnicity, and age. Participants with a caregiver will be given the option whether or not to include their caregiver in the interviews. Participants will be invited to participate in two interviews (refer to Fig. 1): an interview at baseline (within 2–4 weeks of study enrolment), and a follow-up interview towards the end of the study (within 4–6 months of study enrolment).

##### Healthcare providers

All healthcare providers involved in direct patient care at participating DEPs will be sent a letter of information outlining the objectives of the qualitative interviews. A member of the research team will contact those individuals expressing interest, providing additional information as needed and obtaining informed consent.

##### Organizational administrators and health system decision makers

The project coordinator at each DEP will help the research team identify key administrators involved in supporting the implementation of the intervention. The project contact at OTN will identify key health system decision makers involved in the selection, procurement, and implementation of e-Health technologies into the health care system. We will use snowball sampling by asking interview participants in each group to identify other individuals within the organization who are involved in the processes around the selection, procurement, implementation, and ongoing support of e-Health technologies.

#### Data collection

Semi-structured interview guides include questions that cover the following general topics, guided by the principles of Realist Evaluation [37]. Topics include participants’ experiences of learning about and using the technology; changes to health care provider workflow required to effectively use the technology; organizational changes required to support the technology; and health system barriers and facilitators to effective implementation and evaluation (please contact the authors for copies of each respective interview guide). The interview guides were pilot tested on healthcare providers and researchers to ensure clarity and relevance to the overarching research questions. No substantial changes were made to the interview guides during this process. If needed, interview guides will be refined iteratively following individual interviews based on the feedback from participants and the experiences of the interviewers. It is expected interviews will last between 30 and 60 min. Interviews will be audio recorded and transcribed verbatim by an external third party.

#### Data analysis

Written observations and qualitative interviews will be immediately transcribed into word documents and prepared for qualitative analysis. Observations will also be analyzed using thematic analysis strategies [37, 38], identifying key themes that demonstrate important contextual influences and practices related to the implementation and evaluation of the e-Health technologies in actual contexts of health care delivery. A minimum of two reviewers will independently code all transcripts using an open coding process. Following the first five interviews, a coding schema will be created to guide the analysis of subsequent interviews. Open coding will be applied throughout the analysis for content that does not fit within the coding schema. Consolidation of codes and resolution of any disagreements will be achieved through consultation with a third reviewer.

After the thematic analyses of all qualitative data has been completed, the key themes identified will be compared against the implementation and rapid evaluation framework developed in the first phase of the study. The findings of the qualitative data will be used to develop statements of the relationships between (a) key contextual factors, (b) the mechanisms by which they affect the implementation of the e-Health interventions, and (c) the impact on the outcomes of the intervention themselves (in Realist Evaluation these statements are referred to as “Context-Mechanism-Outcome Configurations”) [37]. These statements will then be used to revise the framework in order to more accurately reflect the key contextual influences and practices that constitute the implementation process in actual health care settings in Ontario.

### Limitations

Aspects of the study design limit the generalizability of the study results, including a short follow-up period and the restriction of participant sampling to DEPs, where complexity of patients with T2DM may be lower. In addition, effectiveness cannot be generalized to other relevant clinical outcomes beyond glycemic control (e.g., blood pressure and lipid levels). Required proficiency in the English language limits generalizability beyond this cohort of individuals and the pragmatic nature of this trial. Study participants receive a unique smartphone to enable participation in the study, which may be unfamiliar and pose a challenge when trying to integrate its use into established routines; however, in contrast, providing a unique device to study participants also increases intervention access for those individuals who do not currently own a smartphone.

If the provision of phones is not included in potential plans to scale-up the intervention (if successful), the pragmatic nature of this trial and its generalizability are limited. Furthermore, as cost estimates will not include the cost of the phones and additional staff required to implement the intervention, subsequent scale-up activities need to acknowledge this limitation by including these costs in their scale-up models or assuming a natural penetration estimate for smartphone use in a given clinical population. As a study-specific phone was provided to all participants, those already in possession of a phone were required to utilize both phones, which may negatively impact the ecological validity. As a result, the impact of carrying two phones will be explored in the qualitative evaluation. The mobile application was developed in an American context with different guideline recommendations, which may influence its utility and effectiveness in a Canadian context. Finally, it is uncertain how findings will be able to be generalized from this specific evaluation to apply to other mobile applications design to improve self-management for individuals with T2DM.

## Discussion

Web and mobile-based platforms for self-management interventions are promising as they can improve patient knowledge, and clinical outcomes [39] while being scaled to improve access for patients who are computer-literate at little cost [40]. This is particularly important for providing care for individuals with diabetes, where self-management plays a pivotal role to improve health outcomes. Mobile-based interventions to enhance self-management provide increased flexibility, convenience, and patient access [32] and leverages the fact that three out of four Canadians own a personal smartphone [41].

Results of this trial will provide much needed information about the clinical- and cost-effectiveness of the mobile application. As the application was developed and evaluated in the United States, this study aims to evaluate the application’s potential to improve diabetes self-management in the Canadian context. The process evaluation fills a gap in the literature, providing valuable insight into the contextual factors that influence the effectiveness of the application. The results of the process evaluation will inform considerations around whether and how to scale-up the intervention system-wide and enhances the translational aspect of this work. This aspect of the evaluation will have significant implications for healthcare administrators, government agencies, and other stakeholders interested in the use of e-Health technologies to improve health status and patient experience alongside system-level outcomes.

### Trial status

The trial is actively recruiting participants across all three sites. A total of 14 participants have been recruited to date.

## Abbreviations

AHRC: Applied health research centre
ANCOVA: Analysis of covariance
CRF: Case report form
DEP: Diabetes education program
ICES: Institute for clinical evaluative sciences
ITG: Immediate treatment group
MCID: Minimally clinical important difference
ODB: Ontario drug benefit
OHIP: Ontario health insurance plan
OTN: Ontario telemedicine network
PAID 5: Problem areas in diabetes
PHIPPA: Personal health information protection act
PREM: Patient-reported experience measure
PROM: patient-reported outcome measure
SDSCA-6: Summary of diabetes self-care activities
T2DM: Type 2 diabetes
WLC: Wait list control
